# Trends in Epidemiology: Analysis of Risk Factors and Outcomes of Infective Endocarditis: A Retrospective Study (2009–2015)

**DOI:** 10.7759/cureus.3910

**Published:** 2019-01-17

**Authors:** Aung Naing Lin, Htoo Kyaw, Kyawzaw Lin, Sima Pendharkar, Atif Z. Shaikh, Cesar Ayala-Rodriguez, Joseph Abboud, Sarath Reddy

**Affiliations:** 1 Internal Medicine, Brooklyn Hospital Center/Mount Sinai Hospital, New York, USA; 2 Cardiology, Brooklyn Hospital Center/Mount Sinai Hospital, New York, USA

**Keywords:** infective endocarditis (ie), health care associated infections, african americans, cardiology

## Abstract

Background

Despite advanced diagnosis and treatment, infective endocarditis (IE) is a potentially life-threatening condition. Although recent studies have provided evidence of changing trends in IE epidemiology, few studies examine patterns within urban minority populations. Here we present the epidemiology, risk factors, and outcomes of IE among an underserved African American population in Brooklyn, New York, compared to the general population.

Methods

This is a retrospective study which included 67 patients with IE diagnosed at The Brooklyn Hospital Center from 2009 to 2015. Patients were selected according to the modified Duke Criteria for definite IE. Various epidemiological parameters were examined via chi-square and Fisher’s exact test using SPSS 24 software (IBM Corp., Armonk, NY).

Results

The mean age of the 67 patients was 63 years and 46.3% of the patients were men. The majority of patients (70.1%) were African American while Hispanics and Caucasians were 17.9% and 7.5%, respectively. Healthcare-associated IE (58.2%, n=39) outnumbered community-acquired IE (41.8%, n=28). The sites of vegetation were the mitral valve (62.7%, n=42), tricuspid valve (22.4%, n=15), aortic valve (11.9%, n=8), and intravenous catheter (3%, n=2). In valves, 13.4% of the cases were found in prosthetic valves while the majority occurred in native valves. The most common pathogens of IE were the Staphylococcus (50.8%, n=34) species, followed by Streptococcus species (32.8%, n=22). Overall, the in-hospital mortality was 38.8% (n=26) with higher mortality observed for healthcare-associated IE than community-acquired IE (P = .049). Embolic complications were associated with significant mortality (P < .001).

Conclusion

Our study demonstrated that the common causative pathogens for IE among African Americans trends towards Staphylococcus species followed by Streptococcus species, similar to the contemporary epidemiology of IE. Healthcare-associated IE outnumbered community-acquired IE and was associated with higher mortality. Embolic complications were significantly associated with high mortality. Therefore, efforts made to control healthcare-associated infections are expected to decrease the trend of IE.

## Introduction

Infective endocarditis (IE) is a life-threatening disease characterized by infection of the endocardial surface of the heart. IE commonly affects the mitral valve, followed by the aortic, tricuspid, and pulmonary valve but can also involve supporting structures and any part of the mural endocardium. Although numbers for rheumatic fever and rheumatic heart disease have been trending down over the last decades, the epidemiology of IE has experienced substantial changes especially in causative organisms and drug-resistant organisms. This could be a part of an increasing elderly population and the emergence of new risk factors including intracardiac or intravenous devices, immunosuppressive conditions such as diabetes, hemodialysis, and intravenous drug use [1-6]. Complications of IE not only affect the valvular structures and function of the heart but also manifest as systemic embolization, mainly to the central nervous system [7]. Despite advanced diagnostic and therapeutic options, the morbidity and mortality of IE have not decreased in recent decades. Currently, IE ranks as the third or fourth most life-threatening infection following sepsis, pneumonia, and intra-abdominal abscess [8-10]. Epidemiology studies of IE have been conducted worldwide, but few studies have represented urban minorities [1,3,5,6]. The main purpose of this study is to examine the temporal trend in the epidemiology, microbiology, management, and complications of IE among an underserved African American population in an urban setting in comparison to the general population.

## Materials and methods

We performed a retrospective analysis of all patients above 18 years of age admitted to The Brooklyn Hospital Center from January 2009 to December 2015 where IE was confirmed by transthoracic echocardiogram (TTE) or transesophageal echocardiogram (TEE) and blood culture according to modified Duke Criteria for definite IE (two major criteria or one major criterion and three minor criteria or five minor criteria). Clinical data were obtained from a thorough review of charts and electronic medical records of The Brooklyn Hospital Center which is a safety net hospital that primarily serves African American populations. All available variables including patient demographics, ethnicity, comorbid conditions, preexisting valvular status, type of causative organism including sensitivity and resistance to antibiotics, blood culture reports, site, size, number of vegetations, structural and functional manifestations by echocardiography, systemic complications especially embolic or immunologic phenomena, management outcomes and in-hospital mortality were examined and correlated with multidisciplinary experts’ opinions. Next, patients were subclassified into community-acquired IE or healthcare-associated IE to encompass both acute endocarditis and subacute endocarditis. Healthcare-associated IE was defined as endocarditis in those residing in long-term care facilities including nursing home residents, patients undergoing hemodialysis or peritoneal dialysis, and immunosuppressed or immune-compromised patients such as those with human immunodeficiency virus (HIV) infection, underlying malignancies, transplant recipients, corticosteroid use and receiving chemotherapy or radiation therapy, and those who were hospitalized for more than 48 hours or were rendered healthcare services in the past 45 days.

The collected data were compiled and analyzed with the aid of IBM SPSS Statistics for Windows, Version 24.0 (IBM Corp., Armonk, NY). The proportions of categorical variables were examined and then further analyzed using chi-square analysis and Fisher’s exact test. All the reported associations were two-sided and assumed significant if the P < .05. The mean and standard deviation were calculated for continuous variables.

## Results

Epidemiology

A total of 67 patients were found to have definite IE based on modified Duke's criteria from January 2009 to December 2016. Of these, 47 patients (70.1%) were African American, 12 patients (17.9%) were Hispanic, and five patients (7.5%) were Caucasian; this was followed by Asian and others ethnicities contributing less than 5% in total. The patients’ mean age was 63 years. Seven cases (10.4%) occurred in those 20–50 years of age and 60 cases (89.6%) developed in those over 50 years of age; among these, 46.3% were above 70 years old. The gender distribution was nearly proportionate with the female-to-male ratio being 1.16 as there were 36 women (53.7%) and 31 men (46.3%) involved in the study.

Of the studied patients, 77.6% (n=52) were newly diagnosed with IE, and 22.4% (n=15) had a previous history of IE. Participants were subclassified into community-acquired (41.8%, n=28) and healthcare-associated (58.2%, n=39). The most common underlying comorbid conditions were recorded as diabetes mellitus (32.8%, n=22), arrhythmia (23.9%, n=16), hypertension (17.9%, n=12), and dyslipidemia (10.4%, n=7) while 14.9% (n=10) had no known co-morbidity. One-third of all patient required long-term hemodialysis (28.4%, n=19) for underlying end-stage renal disease. Among the studied population, a history of intravenous drug abuse in 11 patients (16.4%) and HIV in eight patients (11.9%) was recorded. A total of 38 patients (56%) were predisposed to valvular regurgitation not related to rheumatic heart disease, and 28 patients (41.8%) had intra-cardiac devices. Only one patient was found to have congenital heart disease.

Microbiology

Table 1 demonstrates all the common, rare and unusual pathogens causing IE in our patient population in detail. Both gram-positive (85.2%, n=57) and gram-negative microorganisms (8.8%, n=6) were detected while other organisms (6%, n=4) were unspecified by gram stain and were fungal infections. The gram-positive organism mainly consisted of Staphylococcus species (50.8%, n=34) and Streptococcus species (32.9%, n=22) whereas one rare and unusual case of Erysipelothrix rhusiopathiae was recognized. Of the Staphylococcus aureus (n=25), half (n=13) were identified as methicillin resistant. Additionally, Staphylococcus epidermidis (n=8) and Staphylococcus lugdunensis (n=1) were Staphylococcus species involved. Enterococcus faecalis (n=9), Viridans streptococcus (n=3), Group B-beta hemolytic streptococcus (n=5), and Streptococcus pneumoniae (n=3) were among the Streptococcus species while Abiotrophia (n=1) and Aerococcus species (n=1) were unusual species observed. Nonetheless, the most common isolated organism were Staphylococcus aureus (37.4%, n=26) followed by Enterococcus faecalis (13.4%, n=9), Staphylococcus epidermidis (11.9%, n=8), Streptococcus viridans (4.5%, n=3), and culture negative organisms (4.5%, n=3). Other rare gram-negative organisms included Serratia marcescens, Klebsiella pneumoniae, Pseudomonas aeruginosa, and Escherichia coli. No HACEK (Haemophilus species, Aggregatibacter species, Cardiobacterium hominis, Eikenella corrodens, and Kingella species) group bacteria were identified in our study groups.

**Table 1:** All causative organisms in the study population

Causative Organism	Percentage (%)	Number (n)
Staphylococcus species	50.8%	34
Staphylococcus aureus	37.4%	25
Staphylococcus epidermidis	11.9%	8
Staphylococcus lugdunensis	1.5%	1
Streptococcus species	32.9%	22
Enterococcus faecalis	13.4%	9
Viridans streptococci	4.5%	3
Group B beta hemolytic Streptococcus	7.5%	5
Streptococcus pneumoniae	4.5%	3
Abiotrophia species	1.5%	1
Aerococcus species	1.5%	1
Rare gram-positive organisms		
Erysipelothrix rhusiopathiae	1.5%	1
Gram-negative organisms	8.8%	6
Serratia marcescens	2.9%	2
Klebsiella pneumoniae	2.9%	2
Pseudomonas aeruginosa	1.5%	1
Escherichia coli	1.5%	1
Fungal and others	6%	4
Candida parapsilosis	1.5%	1
Culture negative organisms	4.5%	3

Skin infection (52.2%, n=35), oral or gingival (31.3%, n=21), and minor procedures (16.4%, n=11) were identified as common modes of entry. The most common site of vegetation was the mitral valve (62.7%, n=42) followed by the tricuspid valve (22.4%, n=15), aortic valve (11.9%, n=8) and intravenous catheter (3%, n=2). However, no pulmonary valve vegetation was localized. Involvement of native valves and prosthetic valves accounted for 86.6% (n=58) and 13.4% (n=9) of the cases, respectively. No significant correlation between organisms and mode of entry or site of vegetation was found.

TTE was able to localize vegetations in 46.3% (n=31) of cases whereas TEE was required in 53.7% (n=36). A single vegetation was identified in 58 patients (86.6%) among which 68.7% were less than 1 cm and 31.3% were more than 1 cm in size. Only nine patients (13.4%) were found with multiple vegetations. Blood cultures were obtained before the initiation of antibiotics in all patients, but only 46.3% (n=31) were returned as positive. There was no observed correlation between the causative organisms and the size or numbers of vegetations.

Management outcomes and mortality

All the patients involved met systemic inflammatory response syndrome or sepsis criteria at presentation. Among the patients, 38.8% (n=26) received conservative management with antibiotics while 61.2% (n=41) required intervention—either a vegetectomy or valve replacement; among these 48.8% (n=20) died before undergoing surgical intervention. Only 19.2% (n=5) of the IE patients who were managed with antibiotics expired, in comparison to those who required surgery and died before intervention. The association between management options and mortality was significant (P = .009 by Pearson chi-squared test and corrected P = .011 by Fisher's exact test). Of the patients managed with antibiotics, 26.9% (n=7) had associated embolic manifestations. On the other hand, 81.6% (n=31) of patients requiring intervention were complicated with either major or minor embolic events before intervention. Similarly, embolic events were associated with significant mortality (P < .001 by both Pearson chi-square and Fisher's exact test) as there was a 68.4% (n=26) mortality rate in patients with embolic complications such as central nervous system embolism, peripheral embolism and/or coronary embolism.

Overall in-hospital mortality was investigated and found to be 38.8% (n=26), and patients in all instances experienced embolic complications. There was higher mortality in healthcare-associated (73.1%, n=19) than community-acquired (26.9%, n=7) IE; the associations were weakly significant (P = .049). In a cross-tabulation of gender and mortality, half of all female participants (n=18) died compared with 25.8% of male participants (n=8). It can be assumed that women had a higher mortality rate than their male counterparts but the association was weak (P =.43 by Pearson chi-square and corrected P = .49 by Fisher's exact test).

## Discussion

There are limited studies specifically representing urban African American minorities and patterns with regards to endocarditis given that as previous studies focused on the general US population. The salient points of our study are that we focused on an underserved minority African American population in Brooklyn. We examined the epidemiology trends and compared all parameters including age, sex, pathogens, underlying co-morbidities, pre-disposing factor, management options, complications and in-hospital mortalities for healthcare-associated IE and community-acquired IE.

Of the Brooklyn patient population with IE, 70.1% were African American. The majority of patients were over 50 years of age; only 10.4% were 20 to 50 years old. The mean patient age was 59 years, approximately equal to the mean age of 57.9 years in the International Collaboration on Endocarditis-Prospective Cohort Study (ICE-PCS) in 2009 and the systemic review conducted by Slipczuk et al. in 2013 [3,11]. This is higher than the mean age of 45.3 years in the 1980s probably because of the growing numbers of the adult population and frequent use of intracardiac and other medical devices [3]. Though we did not calculate the incidence of IE in our study, it was estimated to be higher than the mean national average because the study population was underserved medically and of poor socioeconomic status (SES).

Overall, Staphylococcus species were the most common, accounting for 50.7% of all cases, outnumbering the 32.8% of cases involving Streptococcus species. The most commonly isolated pathogens were Staphylococcus aureus and Staphylococcus epidermidis, involved in 38.85 and 11.9% of all cases, respectively. Methicillin-resistant Staphylococcus aureus was isolated in 13 of 25 patients with Staphylococcus aureus IE. The trend in causative organisms of IE in our study was similar to that of current trends in developed countries. These results demonstrate that trends for IE in the African-American minority in Brooklyn follow the current national and global trends as highlighted by other studies in the past decades [1-7,11-12].

However, among the Streptococcus species found in the study, Enterococcus were more common than Viridians group streptococcus. No HACEK group organism was identified in our study. The only fungal-induced IE was one case of lethal Candida parapsilosis. Additionally, three cases of culture-negative endocarditis were detected. Others rare and unusual organisms that contributed to one-quarter of the cases found in our study included Erysipelothrix rhusiopathiae, Aerococcus, Abiotrophia species, Group B beta-hemolytic Streptococcus pneumoniae, Serratia marcescens, Klebsiella pneumoniae, Pseudomonas aeruginosa, and Escherichia coli. The association between causative organisms and sex, age, the status of intravenous drug use, history of HIV or AIDS, hemodialysis, size or site of vegetation, complications or mortality was examined and was found to be statistically non-significant. This may be due in part to either underlying comorbid conditions and poor SES or an increasing number of rare and unusual organisms causing IE among the studied minority population. More thorough prospective research is needed to prove the actual IE trends due to rare and unusual organisms.

Another interesting finding was that the number of cases of healthcare-associated IE (58.2%, n=39) outnumbered those community-acquired (41.8%, n=28); this is discordant with the estimated 38.1% for North America by ICE-PCS in 2009 [11]. There was a weak association between healthcare-associated IE in-hospital mortality (P=.049).

The most common site of vegetation was the mitral valve (62.7%, n=42) in agreement with current trends. No significant association between the location of vegetation and either higher in-hospital mortality rate or higher rate of complications was demonstrated. Embolic complications such as central nervous system embolism, peripheral embolism and/or coronary embolism were more common, and embolic events were associated with significant mortality (P< .001) other than heart failure.

Overall in-hospital mortality was investigated and found to be 38.8%. Of these cases, all patients experienced embolization. Although women had a weakly significant increased rate of mortality than men (P=.43 by Pearson chi-square and corrected P=.49 by Fisher's exact test), no significant correlation in between a specific organism, underlying co-morbid conditions, and vegetation size or number was identified in this study.

The association between choice of management options and mortality was significant (P=.009). Although the association between management option and mortality was statistically significant, there might be some confounding factors influencing this association. For example, some patients who really in need of intervention died prior to or while preparing or optimizing for the intervention or were unable to undergo procedures due to deterrence of advanced age or underlying medical conditions. Moreover, the need for transfer to a cardiothoracic surgery facility was an additional factor to consider.

## Conclusions

The common causative pathogens among African Americans trend towards Staphylococcus species followed by Streptococcus species, similar to the contemporary epidemiology of IE. Cases of healthcare-associated IE exceeded community-acquired IE and have relatively higher mortality. Therefore, efforts made to control healthcare-associated infections will favor reducing the overall trend in IE. Embolic complications have been significantly associated with high mortality, and delayed intervention leads to fatality.
